# A novel resting-state functional magnetic resonance imaging signature of resilience to recurrent depression

**DOI:** 10.1017/S0033291716002567

**Published:** 2016-11-08

**Authors:** C. I. Workman, K. E. Lythe, S. McKie, J. Moll, J. A. Gethin, J. F. W. Deakin, R. Elliott, R. Zahn

**Affiliations:** 1 The University of Manchester & Manchester Academic Health Sciences Centre, Institute of Brain, Behaviour and Mental Health, Neuroscience & Psychiatry Unit, Manchester, UK; 2 The University of Manchester & Manchester Academic Health Sciences Centre, School of Psychological Sciences, Neuroscience and Aphasia Research Unit, Manchester, UK; 3 Cognitive and Behavioral Neuroscience Unit, D'Or Institute for Research and Education (IDOR), Rio de Janeiro, RJ, Brazil; 4 Department of Psychological Medicine, Institute of Psychiatry, Psychology, and Neuroscience, Centre for Affective Disorders, King's College London, London, UK

**Keywords:** Biomarkers, depression, frontal lobes, longitudinal studies

## Abstract

**Background:**

A high proportion of patients with remitted major depressive disorder (MDD) will experience recurring episodes, whilst some develop resilience and remain in recovery. The neural basis of resilience to recurrence is elusive. Abnormal resting-state connectivity of the subgenual cingulate cortex (sgACC) was previously found in cross-sectional studies of MDD, suggesting its potential pathophysiological importance. The current study aimed to investigate whether resting-state connectivity to a left sgACC seed region distinguishes resilient patients from those developing recurring episodes.

**Method:**

A total of 47 medication-free remitted MDD patients and 38 healthy controls underwent resting-state functional magnetic resonance imaging (fMRI) at baseline. Over 14 months, 30 patients remained resilient whilst 17 experienced a recurring episode.

**Results:**

Attenuated interhemispheric left-to-right sgACC connectivity distinguished the resilient from the recurring-episode and control groups and was not correlated with residual depressive symptoms.

**Conclusions:**

The current study revealed a neural signature of resilience to recurrence in MDD and thereby elucidates the role of compensatory adaptation in sgACC networks.

## Introduction

Major depressive disorder (MDD) is recurrent in a large proportion of patients, whilst some patients develop resilience after recovering from a major depressive episode (MDE; American Psychiatric Association, 2000). The neural basis of resilience to recurrent MDEs is poorly understood. There is therefore an urgent need to characterize the neural bases of resilience and, relatedly, vulnerability to recurrence to improve stratification of patients and to identify novel targets for therapeutic interventions. Resting-state functional magnetic resonance imaging (fMRI), frequently used to measure low-frequency fluctuations in blood oxygen-level dependent (BOLD) signals (Fox & Raichle, 2007), is particularly promising for understanding the neural basis of resilience from the perspective of network models of MDD (Seminowicz *et al.* 2004; Price & Drevets, 2010).

Abnormal functional connectivity within subgenual cingulate cortex (sgACC) networks has been demonstrated repeatedly in cross-sectional studies of MDD (Greicius *et al.* 2007; Sheline *et al.* 2010; Gaffrey *et al.* 2012; Herringa *et al.* 2013; Dutta *et al.* 2014) and this region is thought to play a central role in the pathophysiology of MDD (Dunlop & Mayberg, 2014). In a cross-sectional activation fMRI study, our group reported lower functional connectivity between an anterior temporal lobe (ATL) seed region and the sgACC during the experience of guilt (self-blame) relative to indignation (other-blame) in remitted MDD (rMDD) patients compared with a healthy control (HC) group (Green *et al.* 2012). In a subsequent prospective activation fMRI study by our group, functional connectivity between these regions was higher during self-blame in rMDD patients who subsequently developed a recurring episode (Lythe *et al.* 2015) compared with those who remained stable and with a HC group. Taken together, this led to the hypothesis that the lower self-blame-selective ATL connectivity in rMDD patients seen in the first study (Green *et al.* 2012) reflected a signature of resilience rather than vulnerability as was initially thought (Lythe *et al.* 2015). This was based on the observation that the cross-sectional study included a large proportion of MDD patients in full recovery for more than 1 year as well as a large proportion of first-episode patients (Green *et al.* 2012). When investigating ATL–sgACC functional connectivity irrespective of psychological condition (i.e. self-blame *v*. other-blame), however, there was no evidence of abnormalities in either the resilient or the recurring-episode MDD groups (Lythe *et al.* 2015). These activation fMRI data precluded a more systematic investigation of sgACC network connectivity that included regions other than the ATL. This is because for activation fMRI-based connectivity models, the selection of seed regions that show different levels of average activation during the psychological conditions of interest are problematic because of confounding co-activation and connectivity (Friston *et al.* 1997). Since the sgACC region displays higher activation in guilt-prone individuals during self-blame relative to other-blame (Zahn *et al.* 2009*a*, *b*; Green *et al.* 2012), it could not be used as a seed region in our previous activation fMRI-based connectivity studies. In contrast, resting-state fMRI-based connectivity does not suffer from this limitation and is therefore well suited to mapping sgACC networks underpinning resilience more systematically. Furthermore, the acquisition of resting-state fMRI has some important advantages for clinical neuroimaging investigations since scans can be acquired relatively quickly (less than 10 min) and without needing to implement and interpret complex psychological paradigms.

Higher resting-state functional connectivity between the subgenual and posterior cingulate cortices distinguished vulnerable adolescents remitted from preschool-onset MDD from a HC group (Gaffrey *et al.* 2012). Treatment studies using resting-state fMRI in MDD have revealed a relationship between treatment response and pre-treatment connectivity to the sgACC (reviewed in Dichter *et al.* 2014). Whether patterns of sgACC resting-state functional connectivity, however, are distinctly altered in rMDD patients who will remain resilient compared with those who will go on to experience a recurrent MDE remains unknown.

We aimed to address this question by investigating whether resting-state functional connectivity to the sgACC could distinguish medication-free rMDD patients who would remain resilient over a 14-month follow-up period from patients who would go on to experience a recurrent MDE and also from a HC group. It is important to underline that this study enrolled patients recovered from the depressed state and was therefore well suited to identify physiological indices of sustained recovery, referred to here as resilience to recurrent MDEs, but not of resilience in general. Our aims were accomplished using a seed-based approach to analyse resting-state fMRI data acquired at the outset of study participation. The left anterior sgACC seed region was placed using coordinates described by Green *et al*. (2012) and was chosen for its close proximity to subgenual regions implicated in vulnerability to MDD (Green *et al.* 2012; Herringa *et al.* 2013; Workman *et al.* 2016). We predicted that abnormal connectivity of the sgACC with a fronto-subcortical network would distinguish resilient from recurring-episode MDD patients. More specifically, we predicted that lower connectivity of the sgACC would be observed in the resilient MDD patients compared with both the recurring-episode MDD and HC groups. In other words, we predicted that the direction of connectivity in the resilient MDD patients would be the opposite to that reported in currently depressed patients, previously found to demonstrate hyperconnectivity of the sgACC (reviewed in Dutta *et al.* 2014).

## Method

### Participants

This study received approval from the South Manchester National Health Service Research Ethics Committee (reference no. 07/H1003/194) and all participants gave informed consent after the study procedures were explained in full (verbal consent for the telephone-based screening and 3-month follow-up interviews and written consent at the start of each study visit). The authors assert that all procedures contributing to this work comply with the ethical standards of the relevant national and institutional committees on human experimentation and with the Helsinki Declaration of 1975, as revised in 2008. Participants were recruited with online and print advertisements and received compensation for their time and travel expenses as part of the UK Medical Research Council (MRC)-funded ‘Development of Cognitive and Imaging Biomarkers Predicting Risk of Self-Blaming Bias and Recurrence in Major Depression’ project (Lythe *et al.* 2015; Zahn *et al.* 2015). A preliminary assessment of eligibility was conducted via telephone for 707 volunteers (a copy of the screening form is available at http://www.translational-cognitive-neuroscience.org/start/test-materials). The 276 eligible volunteers following the telephone screening were invited to complete a clinical interview overseen by a senior psychiatrist (R.Z.). The 202 participants who agreed to the interview provided clinical and family histories, a urine sample for toxicology screening, and were assessed with the Structured Clinical Interview-I for Diagnostic and Statistical Manual of Mental Disorders, 4th Edition, Text Revision (DSM-IV-TR) (SCID-I) to diagnose past MDEs and to detect current Axis I disorders (moderate to perfect inter-rater reliability; online Supplementary Table S1; American Psychiatric Association, 2000; First *et al.* 2002). Of these, 48 HC participants and 96 rMDD patients were eligible to take part in the present study following the clinical interview. Of the HC participants, 39 subsequently underwent MRI scanning, though imaging data were excluded for one HC participant due to a pituitary abnormality, resulting in a final HC sample of *n* = 38. Of the rMDD patients, 63 underwent MRI scanning, though imaging data were excluded for six patients who did not complete the longitudinal study visits described below, resulting in a final patient sample of *n* = 57.

A detailed overview of the reasons for which participants were excluded is provided in online Supplementary Table S2. Inclusion criteria were: aged 18–65 years, right handed, English spoken as the native language, and normal or corrected-to-normal vision and hearing. Additional inclusion criteria for the rMDD group were: past MDE and MDD diagnosed by a senior psychiatrist (R.Z.) according to DSM-IV-TR criteria (American Psychiatric Association, 2000), International Classification of Diseases 10th Revision-diagnosed past moderate or severe MDE (World Health Organization, 1992), and remission of symptoms at least 6 months prior to enrollment. Of note, the majority of MDD patients enrolled into this study had previously responded to psychological interventions or first-line antidepressants, with only a small fraction of patients having previously received treatment with second-line antidepressants (see Table 1). The MDD group was therefore predominantly comprised of patients with good treatment response, such as those seen in primary care, rather than the treatment-resistant patients typically seen in secondary care. Exclusion criteria were: current or relevant past Axis I disorders (e.g. history of substance abuse), psychotropic medication use within 4 weeks of enrollment (8 weeks for fluoxetine), acute suicidality/self-harming behaviours, impaired psychosocial functioning measured with the Global Assessment of Functioning scale (American Psychiatric Association, 2000), a Montgomery–Åsberg Depression Rating Scale (MADRS) score >10 (Montgomery & Åsberg, 1979; Zimmerman *et al.* 2004), history of neurological or medical disorders affecting brain functioning, developmental disorders or learning disabilities, an Addenbrooke's Cognitive Examination score <88 (conducted in participants aged over 50 years; Mioshi *et al.* 2006), and contraindications for MRI scanning. Additional exclusion criteria for the HC group were: history of Axis I disorders, first-degree family history of mood disorders or schizophrenia.

**Table 1.** Clinical characteristics of the recurring-episode and resilient MDD patients^a^

	Recurring episode MDD (*n* = 17)	Resilient MDD (*n* = 30)
Past MDD subtype, *n*		
With melancholic features	9	17
With atypical features	0	2
No specific subtype	8	11
Number of previous MDEs, *n*		
1	1	13
2	5	5
3	2	9
4	4	1
5	3	2
6 or more	2	0
Average number of previous MDEs*	3.7 (2.0, 1–9)	2.1 (1.2, 1–5)
Last and most severe MDE details		
Average length of MDE, months	17.7 (25.2, 1–96)	15.3 (18.8, 1–81)
Average time in remission, months	21.5 (20.9, 6–72)	37.9 (53.8, 6–282)
Average MADRS score for MDE	34.6 (5.2, 24–44)	35.1 (5.7, 20–44)
No psychotropic medication since, months	42.7 (54.4, 2–173)	60.0 (86.5, 3–372)
Previous treatment, *n*		
SSRI antidepressant	15	25
SNRI antidepressant	0	1
Tricyclic antidepressant	0	2
Mirtazapine	1	0
Unknown class of antidepressant	3	3
Benzodiazepines only	0	1
No antidepressant medication	1	2
CBT	6	6
Self-guided CBT via Internet, books	0	3
Counselling	6	14
Suicide attempts	0.18 (0.53, 0–2)	0.20 (0.61, 0–3)
Lifetime Axis I co-morbidity^b^, *n*		
Panic disorder with agoraphobia	1	0
Bulimia nervosa	0	1
No lifetime co-morbidity	16	29
Family history, *n*		
First-degree relative with MDD	10	16
No family member with history of MDD	6	11
First-degree relative with schizophrenia or bipolar disorder	1	3

Data are given as mean (standard deviation, range) unless otherwise indicated.

MDD, Major depressive disorder; MDE, major depressive episode; MADRS, Montgomery–Åsberg Depression Rating Scale; SSRI, selective serotonin reuptake inhibitor; SNRI, serotonin norepinephrine reuptake inhibitor; CBT, cognitive–behavioural therapy.

a All MDD patients stopped medication before the required washout phase. Recurring-episode and resilient MDD patients did not significantly differ on past MDD subtype, average length of the last MDE, average time in remission, average MADRS score for the last MDE, average time since last taking psychotropic medications, number of patients previously treated, number of suicide attempts, lifetime Axis I co-morbidity, or family history (contingency coefficient < 0.20, *p* > 0.18; *t* < 1.21, *p* > 0.23). There were also no differences between the resilient and recurring-episode MDD patients regarding previous treatment with SSRIs, SNRIs, tricyclics, mirtazapine or CBT (contingency coefficient < 0.20, *p* > 0.17).

b All co-morbid disorders were fully remitted at the time of study and none was likely to be the primary cause of the depressive episodes.

* Significantly different between the recurring-episode and resilient MDD groups (*t*_45_ = 3.39, *p* = 0.001).

The rMDD patients completed follow-up interviews via telephone or in person at 3, 6 and 14 months after enrollment using the MDD module and psychosocial functioning assessment from the Longitudinal Interval Follow-up Evaluation interview for DSM-IV (LIFE-IV; Keller *et al.* 1987). The LIFE interview includes a six-point Psychiatric Status Rating (PSR): (1) no residual symptoms; (2) one or more mild symptoms causing no relevant distress or impairment; (3) mild symptoms causing no more than moderate distress or impairment; (4) major symptoms not meeting full criteria for an MDE; and (5–6) major symptoms meeting criteria for an MDE. The raters were trained by the creators of the LIFE interview and inter-rater reliability was excellent (online Supplementary Table S1). Importantly, participation in the current study ended when patients developed an MDE. Of the 57 rMDD patients who completed the study, 30 remained in stable remission (resilient MDD group), 17 experienced a recurrent MDE (i.e. at least one MDE during the 14-month follow-up period; recurring-episode MDD group), and 10 developed symptoms not meeting full criteria for an MDE (i.e. a PSR of 3 requiring treatment or a PSR of 4; subthreshold symptom group). The analyses presented below include the resilient and recurring-episode MDD groups, but exclude the subthreshold symptom group.

The resilient MDD, recurring-episode MDD and HC groups were well-matched on demographic variables (Table 2). The resilient and recurring-episode MDD groups did not differ from the HC group on age, sex or years of education. Compared with the HC group, however, scores on the Beck Depression Inventory (BDI; Beck *et al.* 1996) were higher in both the resilient (*t*_66_ = 2.96, *p* = 0.004) and recurring-episode MDD groups (*t*_53_ = 4.72, *p* < 0.0001). BDI scores were also higher for the recurring-episode MDD group compared with the resilient MDD group (*t*_45_ = 2.22, *p* = 0.03). Nevertheless, average BDI scores for all groups were below 10, suggesting the presence of only minimal subthreshold depressive symptoms (Beck *et al.* 1988). Additionally, no group differences were observed for current scores on the MADRS. The resilient MDD group did not differ from the recurring-episode MDD group on age, sex, education, past MDD subtype, average length of last MDE, months since remission, severity of the last MDE measured with the MADRS, months since last psychotropic use, number of patients previously treated, number of suicide attempts, or family history of MDD. The recurring-episode MDD group did, however, have a greater number of previous MDEs compared with the resilient MDD group (*t*_45_ = 3.39, *p* = 0.001).

**Table 2.** Demographic variables in the recurring-episode and resilient MDD patients and HC group^a^

	Recurring-episode MDD (*n* = 17)	Resilient MDD (*n* = 30)	HC (*n* = 38)
Age, years	35.9 (12.4)	37.6 (12.7)	36.2 (13.8)
Duration of education, years	16.4 (2.6)	17.4 (2.0)	16.8 (2.3)
BDI score*	5.2 (5.0)	2.6 (2.9)	0.9 (1.7)
MADRS score	0.9 (1.7)	0.8 (1.4)	0.7 (1.3)
Sex, *n*			
Male	6	12	13
Female	11	18	25
Frame-wise displacement, mm	0.26 (0.14)	0.24 (0.15)	0.24 (0.15)

Data are given as mean (standard deviation) unless otherwise indicated.

MDD, Major depressive disorder; HC, healthy control; BDI, Beck Depression Inventory; MADRS, Montgomery–Åsberg Depression Rating Scale.

a With the exception of BDI scores, the recurring-episode MDD patients and HC group did not significantly differ on the demographic variables (contingency coefficient < 0.02, *p* > 0.93; *t* < 0.62, *p* > 0.53). Also with the exception of BDI scores, the resilient MDD patients and HC group did not significantly differ on the demographic variables (contingency coefficient < 0.06, *p* > 0.62; *t* < 1.05, *p* > 0.30). Again, with the exception of BDI scores, the recurring-episode and resilient MDD patients did not significantly differ on the demographic variables (contingency coefficient < 0.05, *p* > 0.74; *t* < 1.41, *p* > 0.16).

* Significantly different between the recurring-episode MDD and HC groups (*t*_53_ = 4.72, *p* < 0.0001), between the resilient MDD and HC groups (*t*_66_ = 2.96, *p* = 0.004), and between the recurring-episode and resilient MDD groups (*t*_45_ = 2.22, *p* = 0.03).

### Image acquisition

MRI data were acquired on a 3 T Philips Achieva scanner (Philips Medical Systems, the Netherlands) with an eight-channel coil. A resting-state echo-planar image (EPI) was acquired for each participant using a sequence optimized for detecting ventral frontal signals (240 volumes; 40 axial slices; 3 mm slice thickness; ascending sequential acquisition; repetition time: 2000 ms; echo time: 22 ms; field of view: 240 × 240 × 120 mm; acquisition matrix: 80 × 80 voxels; reconstructed voxel size: 3 mm^3^; flip angle: 90°). Participants were asked to lie motionless with eyes closed during the scan and were debriefed afterwards to confirm the instructions were followed, at which point we confirmed that no participants had fallen asleep. A three-dimensional T1-weighted magnetization-prepared rapid-acquisition gradient-echo (MPRAGE) structural image was also acquired for each participant (160 axial slices; 0.9 mm slice thickness; repetition time: 8.4 ms; echo time: 3.9 ms; field of view: 240 × 191 × 144 mm; acquisition matrix: 256 × 163 voxels; reconstructed voxel size: 0.94 × 0.94 × 0.9 mm; flip angle: 8°). In order to rule out clinically significant neurological abnormalities, T2-weighted structural images were also acquired.

### Resting-state fMRI analysis

The pre-processing pipeline for the resting-state fMRI data has been described in detail elsewhere (Workman *et al.* 2016). Briefly, pre-processing was performed using SPM8 (http://www.fil.ion.ucl.ac.uk/spm/) for compatibility with the DPARSF Advanced Edition (Chao-Gan & Yu-Feng, 2010; http://rfmri.org/DPARSF) and Artifact Detection Tools (ART; http://web.mit.edu/swg/software.htm) MATLAB (MathWorks) toolboxes used in subsequent steps. For each EPI, the first 10 volumes were discarded, then slice timing and head motion correction were performed, and then regressors were created for high-motion volumes using ART (frame-wise signal intensity >3 s.d.s from the global mean, frame-wise head displacement >1 mm). Next, the MPRAGE images were co-registered to the EPIs and segmented, then linear detrending and nuisance covariates regression were performed on the EPIs [24 motion parameters (Friston *et al.* 1996), white matter and cerebrospinal fluid signal, ART regressors], and then the EPIs were normalized with parameters derived during segmentation. After this, the EPIs were smoothed with a 6 mm kernel and band-pass filtered to preserve frequencies between 0.01 and 0.08 Hz. High motion volumes identified by ART were then removed, as were sections of data spanning fewer than five contiguous volumes. All resulting EPIs contained at least 5 min of data (150 volumes).

For each EPI, the average time course within a left anterior sgACC seed region was correlated with the time course of all other brain voxels, resulting in seed-based functional connectivity maps for each participant. The left anterior sgACC was chosen as the seed region because it was previously implicated in connectivity studies of rMDD patients [Montreal Neurological Institute (MNI) coordinates: −4, 23, −5; 6 mm sphere; Green *et al.* 2012; Lythe *et al.* 2015; Workman *et al.* 2016], it is in close proximity to an anterior sgACC region which demonstrated abnormal resting-state functional connectivity in children vulnerable to MDD (MNI coordinates: 2, 23, −6; Herringa *et al.* 2013) and it is close to sgACC regions which demonstrate hyperconnectivity in current MDD patients (Dutta *et al.* 2014). The resulting seed-based functional connectivity maps were then Fisher *Z*-transformed to improve normality.

Next, we conducted a voxel-wise analysis of variance (ANOVA) to compare the seed-based functional connectivity maps from the resilient MDD, recurring-episode MDD and HC groups. Since we sought to identify a main effect of group, the analyses were carried out in SPM12 given that cluster-level family-wise error (FWE) correction of *F* tests cannot be performed in SPM8. We also used seven bilateral *a priori* regions of interest (ROIs) with known structural or functional connections to the sgACC (Vogt & Pandya, 1987; Carmichael & Price, 1996; Kondo *et al.* 2003; Johansen-Berg *et al.* 2008) and which have been implicated in MDD (Elliott *et al.* 2011; Green *et al.* 2012) or social emotional and/or motivational processing (Moll *et al.* 2005; Zahn *et al.* 2009*b*; Elliott *et al.* 2011): the ventromedial prefrontal cortex, anterior temporal cortex, amygdala, hippocampus, septal region, and hypothalamus. A detailed description of the creation of these ROIs has been provided elsewhere (Zahn *et al.* 2009*b*; Workman *et al.* 2016).

Results were considered significant at an uncorrected voxel-level cluster-forming threshold of *p* < 0.001 and a cluster-level FWE-corrected threshold of *p* < 0.05 across the whole brain and *a priori* ROIs. Mean correlation coefficients were extracted from each surviving cluster and entered into a one-way ANOVA with *post-hoc* Bonferroni pairwise comparisons to identify significant group differences in connectivity to the left anterior sgACC, and results were considered significant at *p* < 0.05 (two-tailed).

## Results

### Main effect of group for functional connectivity

**Fig. 1.**
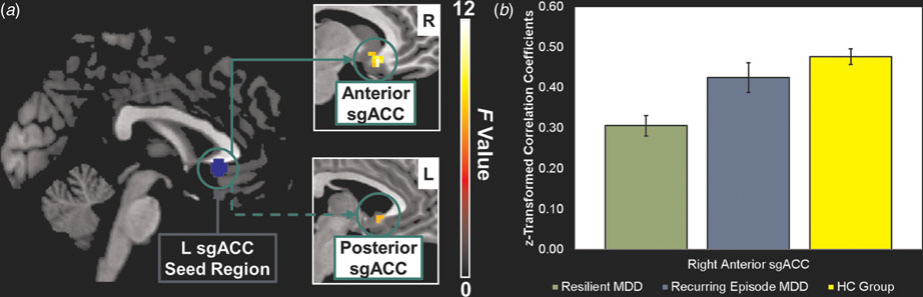
(*a*) Network of regions demonstrating resting-state functional disconnection with the left anterior subgenual cingulate cortex (L sgACC) seed region in the resilient major depressive disorder (MDD) patients. The solid arrow points to regions demonstrating functional disconnection in the resilient MDD patients compared with both the recurring-episode MDD and healthy control (HC) groups. The dashed arrow points to regions demonstrating functional disconnection in the resilient MDD patients compared with the HC group only. Whole-brain images were cropped and displayed at an uncorrected voxel-level threshold of *p* < 0.001. (*b*) Bar plots showing group differences in average *Z*-transformed correlation coefficients and standard errors for the right anterior sgACC cluster. R, Right.

Our analyses revealed a main effect of group (resilient MDD, recurring-episode MDD, HC group) for connectivity of the left anterior sgACC seed region with the right anterior sgACC and with the left posterior sgACC (Table 3; Fig. 1). The main effect of group was further reflected in the extracted cluster averages from both regions (right anterior sgACC: *F*_2,82_ = 14.0, *p* < 0.0001; left posterior sgACC: *F*_2,82_ = 8.7, *p* < 0.0004). Subsequent *post-hoc* Bonferroni-corrected pairwise comparisons showed lower connectivity between the seed region and the right anterior sgACC in the resilient MDD group (mean = 0.31, s.d. = 0.14) compared with both the HC group [mean = 0.48, s.d. = 0.12, *p* < 0.001, mean difference = −0.17, 95% confidence interval (CI) −0.25 to −0.09, *d* = 1.30] and the recurring-episode MDD group (mean = 0.42, s.d. = 0.15, *p* = 0.01, mean difference = −0.12, 95% CI −0.22 to −0.02, *d* = 0.76). In contrast, connectivity between the seed region and this right anterior sgACC region did not differ between the recurring-episode MDD group (mean = 0.42, s.d. = 0.15) and the HC group (mean = 0.48, s.d. = 0.12, *p* = 0.55, mean difference = −0.05, 95% CI −0.15 to 0.04, *d* = 0.44). A different pattern emerged for the left posterior sgACC region which, although showing lower connectivity with the seed region in the resilient MDD group (mean = 0.61, s.d. = 0.22) compared with the HC group (mean = 0.81, s.d. = 0.18, *p* < 0.003, mean difference = −0.20, 95% CI −0.31 to −0.08, *d* = 1.00), showed no difference between the resilient and recurring-episode MDD groups (mean = 0.71, s.d. = 0.19, *p* = 0.29, mean difference = −0.10, 95% CI −0.24 to 0.04, *d* = 0.49). The recurring-episode MDD group (mean = 0.71, s.d. = 0.19) showed no significant differences from the HC group (mean = 0.81, s.d. = 0.18, *p* = 0.26, mean difference = −0.10, 95% CI −0.24 to 0.04, *d* = 0.54) in connectivity between the seed region and this left posterior sgACC region. Therefore, resting-state functional disconnection between the left and right anterior sgACCs, but not between the left anterior and posterior sgACCs, is an abnormality which distinguished the resilient MDD patients from the recurring-episode patients.

**Table 3.** Regions significant for a main effect of group (recurring-episode MDD, resilient MDD, HC group) for functional connectivity to the left anterior subgenual cingulate cortex seed region

Hemisphere	Regions	Peak MNI coordinates			Peak *Z* score	Cluster size	FWE-corrected *p*
		*x*	*y*	*z*			
R	Anterior subgenual cingulate cortex	9	21	−12	4.03	22	0.039^a^^,^^b^
L	Posterior subgenual cingulate cortex	−6	15	−3	3.20	4	0.043^c^^,^^d^

MDD, Major depressive disorder; HC, healthy control; MNI, Montreal Neurological Institute; FWE, family-wise error; R, right; L, left; ROI, region of interest; s.d., standard deviation; CI, confidence interval.

a FWE-corrected at the cluster level over an *a priori* ventromedial prefrontal cortex ROI.

b Lower connectivity with the seed region in the resilient MDD patients (mean = 0.31, s.d. = 0.14) compared with both the recurring-episode MDD (mean = 0.42, s.d. = 0.15, *p* = 0.013, mean difference = −0.12, 95% CI −0.22 to −0.02, *d* = 0.76) and HC groups (mean = 0.48, s.d. = 0.12, *p* < 0.0001, mean difference = −0.17, 95% CI −0.25 to −0.09, *d* = 1.30).

c FWE-corrected at the cluster level over an *a priori* septal region ROI.

d Lower connectivity with the seed region in the resilient MDD patients (mean = 0.61, s.d. = 0.22) compared with the HC group (mean = 0.81, s.d. = 0.18, *p* < 0.0003, mean difference = −0.20, 95% CI −0.31 to −0.08, *d* = 1.00) but not the recurring-episode MDD group (mean = 0.71, s.d. = 0.19, *p* = 0.29, mean difference = −0.10, 95% CI −0.24 to 0.04, *d* = 0.49).

To our knowledge, this is the first study to investigate whether patterns of resting-state functional connectivity are capable of distinguishing between illness courses in young to middle-aged adults with rMDD. As a consequence, it was not possible to conduct *a priori* power analyses based on prior reports. Instead, *post-hoc* power analyses were carried out using the effect sizes reported above at *p* = 0.05 (two-sided). For connectivity between the seed region and right anterior sgACC, we achieved 99.95% power to detect differences between the resilient and HC groups and 68.58% power to detect differences between the resilient and recurring-episode MDD groups. For connectivity between the seed region and left posterior sgACC, we achieved 98.00% power to detect differences between the resilient and HC groups and 34.79% power to detect differences between the resilient and recurring-episode MDD groups.

### Investigation of potentially confounding variables

Next, we investigated whether connectivity between the left and right anterior sgACCs was associated with BDI scores or number of previous MDEs, both of which were elevated in the recurring-episode MDD patients relative to the resilient patients. Across the rMDD patients, however, connectivity between the left and right anterior sgACCs was not associated with BDI scores (*r*_*s*_ = −0.11, *p* = 0.47) or number of previous MDEs (*r*_*s*_ = 0.13, *p* = 0.39). Furthermore, group differences in connectivity between the left and right anterior sgACCs remained significant for the resilient and recurring-episode MDD patients after controlling for the effects of BDI scores (group difference adjusted for BDI scores: *t*_44_ = 3.44, *p* = 0.001) and number of previous MDEs (group difference adjusted for number of previous MDEs: *t*_44_ = 2.61, *p* = 0.01). Importantly, no group differences were observed in frame-wise displacement, a metric of relative head displacement between volumes (Power *et al.* 2012), suggesting the groups were well-matched for head motion (Table 2).

## Discussion

### Main findings and interpretation

Consistent with our general hypothesis, lower connectivity of the left anterior sgACC distinguished resilient from recurring-episode MDD patients. Interestingly, the resilient MDD group showed abnormally low connectivity whilst the recurring-episode MDD patients displayed no difference from the HC group. Intriguingly, we found lower interhemispheric sgACC connectivity to be distinctive of the resilient MDD patients. This pattern of lower functional connectivity was not explained by residual depressive symptoms, which indicates that these results are not neural correlates of incomplete remission. Instead, the pattern of connectivity we have reported is sensitive to aspects of remission not captured by measures of residual symptoms. Furthermore, the recurring-episode MDD patients had more previous MDEs than the resilient patients, as would be predicted by scar theories of depression vulnerability (Burcusa & Iacono, 2007), but number of MDEs was not associated with interhemispheric sgACC connectivity. Our findings therefore confirm the significance of the sgACC to the pathophysiology of MDD by demonstrating for the first time that attenuated interhemispheric sgACC connectivity is associated with resilience to recurrent MDEs.

Patients who are currently in the depressed state have repeatedly been shown to demonstrate increased connectivity to the sgACC that normalizes with treatment (reviewed by Dichter *et al.* 2014; Dutta *et al.* 2014). Findings from studies which investigated resting-state connectivity to the sgACC in populations vulnerable to MDD are less consistent with respect to the direction of abnormal connectivity. For example, Gaffrey *et al*. (2012) described elevated resting-state connectivity between the subgenual and posterior cingulate cortices in patients with a history of preschool-onset MDD. In contrast, Herringa *et al*. (2013) found that lower subgenual cingulate–hippocampal connectivity was associated with a history of childhood maltreatment, a known risk factor for MDD, in otherwise healthy adolescents. Our findings suggest that abnormally low resting-state functional connectivity of the anterior sgACC may reflect a compensatory process in those patients who remain resilient to MDEs, similar to functional compensation mechanisms found in patients with brain lesions (Zahn *et al.* 2006).

The lower interhemispheric sgACC connectivity we observed in the resilient MDD patients may appear to contradict studies which report normalization of resting-state sgACC functional connectivity and cerebral glucose metabolism with treatment (Dichter *et al.* 2014; Dunlop & Mayberg, 2014). These studies typically look at treatment-related changes in recently remitted patients, however, in contrast to the patients studied here who were in stable remission (⩾6 months) at the time of scanning. The risk for experiencing a recurrent MDE is elevated during the first 6 months following remission from the depressed state (Solomon *et al.* 2000). If indeed the abnormally low interhemispheric functional connectivity of the anterior sgACC in resilient MDD patients observed here reflects a compensatory process, this may not emerge until later in the course of recovery. Normal functional connectivity to the anterior sgACC in the recurring-episode MDD patients may reflect a failure to engage, or to continue engaging, this process. Alternatively, connectivity to the sgACC may be linearly associated with depression status, with connectivity to the sgACC ranging from abnormally high in currently depressed patients to abnormally low in patients who remain resilient to recurrent MDEs. Our findings also initially appear inconsistent with our previous interpretation of subgenual cingulate–amygdala resting-state functional disconnection as a primary vulnerability factor for melancholic MDD (Workman *et al.* 2016). However, the pattern of lower subgenual cingulate–amygdala connectivity that we observed in the melancholic MDD patients was independent of vulnerability or resilience to recurring MDEs (see online Supplementary Results). We tentatively interpret this as supportive of our original interpretation of lower subgenual cingulate–amygdala connectivity as a signature of primary vulnerability to melancholia (Workman *et al.* 2016), although this merits further investigation.

To our knowledge, this is the first report of abnormalities in interhemispheric sgACC connectivity in MDD. Clues pertaining to the significance of this finding can be found in reports of psychosurgical interventions for MDD and in lesion studies. The subcaudate tractotomy (and the related limbic leucotomy), in which white matter is lesioned at a site below the caudate and posterior to the orbitofrontal cortex, was historically used to treat chronic MDD with moderate success (Schoene-Bake *et al.* 2010). A tractography study conducted in healthy volunteers with a seed placed in the subcaudate tractotomy lesion site revealed fibre tracts spanning the left and right sgACCs (Schoene-Bake *et al.* 2010), suggesting that disruption of these tracts may be related to clinical improvement in current MDD patients. Relatedly, chronic bilateral deep brain stimulation (DBS) applied to the white matter of the subgenual cingulate cortices in a treatment-resistant MDD group resulted in sustained remission in some patients (Mayberg *et al.* 2005). Although the exact mechanism by which DBS works has yet to be elucidated, the leading explanation is that inhibition occurs at the sites of stimulation (Mayberg *et al.* 2005). Patients with damage to the ventromedial prefrontal cortex, a large swathe of cortex along the medial wall of the frontal lobe which typically encompasses the subgenual cingulate, reported lower depression severity relative to a sample of control participants with damage to other brain regions (Koenigs & Grafman, 2009). Furthermore, damage to the ventromedial prefrontal cortex has been associated with emotional deficits including diminished guilt (Koenigs & Grafman, 2009), which may be excessive or overgeneralized in current MDD patients (American Psychiatric Association, 2000). Taken together, damage to subgenual cingulate white matter pathways and to the ventromedial prefrontal cortices has previously been shown to modulate depressed mood as well as guilt, a distinctive symptom of MDD. The lower interhemispheric anterior sgACC connectivity we have reported in the resilient MDD group relative to the recurring-episode MDD and HC groups is in keeping with these findings.

### Limitations and future directions

The decision to use a seed-based approach to analyse our resting-state fMRI data entailed the selection of an *a priori* ROI which consequently constrained our results. This concern is mitigated, however, by the known importance of the sgACC to MDD as has been detailed throughout. Nevertheless, further functional connectivity investigations are needed to determine whether resting-state networks not detected by our seed-based approach are also associated with resilience to recurrent MDEs. Given that the majority of patients enrolled into this study previously responded to treatment, it is also unclear whether the pattern of interhemispheric sgACC connectivity associated with resilience to recurrence can be generalized to remitted patients with a history of treatment resistance. Future research should seek to validate this signature of resilience to recurrence in patients with varying histories of treatment responsiveness. A general limitation of resting-state fMRI research is that is it not possible to control psychological processes whilst participants undergo scanning. Additional studies are needed to examine the psychological mechanisms underpinning attenuated interhemispheric sgACC connectivity which confers resilience to recurrence. Future longitudinal studies should also aim to replicate these findings and to investigate whether this signature can predict who will develop MDEs in populations without a history of MDD that are nonetheless vulnerable.

### Conclusions

We demonstrated a distinctive pattern of attenuated interhemispheric resting-state sgACC connectivity in MDD patients resilient to recurrence. To our knowledge, this is the first resting-state fMRI signature of resilience to recurrence in patients who are remitted from the depressed state. The pattern of connectivity observed in the resilient MDD patients represents a potential target for therapeutic interventions aimed at improving resilience to future MDEs.

## Supplementary material

For supplementary material accompanying this paper visit http://dx.doi.org/10.1017/S0033291716002567. click here to view supplementary material
