# Supplementary material for: A novel resting-state functional magnetic resonance imaging signature of resilience to recurrent depression
Source: Psychol Med. 2016 Nov 8;47(4):597–607. doi: 10.1017/S0033291716002567 (PMC5426313; doi:10.1017/S0033291716002567)
Supplement: Supplementary file 1 [file S0033291716002567sup001.doc]

**Supplementary material**

**A novel resting-state functional MRI signature of resilience to recurrent depression**

Clifford I. Workman, Karen E. Lythe, Shane McKie, Jorge Moll, Jennifer A. Gethin, John F. W. Deakin, Rebecca Elliott, Roland Zahn

**Supplementary Results**

In an earlier cross-sectional resting-state fMRI study which included the participants studied here, our group found subgenual cingulate-amygdala resting-state functional disconnection to be distinctive of remitted depressed patients with a history of melancholic major depressive episodes (MDE) compared to non-melancholic and healthy control groups . We argued that subgenual cingulate-amygdala functional disconnection is a signature of primary vulnerability for melancholic major depressive disorder (MDD). In view of the present findings suggesting lower interhemispheric connectivity between the subgenual cingulate cortices promotes resilience to recurring MDEs, we wanted to determine whether the network of lower connectivity we previously observed in the melancholic remitted MDD (rMDD) patients is better understood as promoting resilience. To this end, we first extracted the mean Fisher Z-transformed correlation coefficients from the amygdala cluster as described previously for each participant in the current study. These data were then entered into a two-way ANOVA in SPSS 20 with between-subjects factors for group (resilient or recurring episode MDD) and for subtype (melancholic or non-melancholic). Results were considered significant at p<0.05 two-tailed.

For subgenual cingulate-amygdala resting-state connectivity, we observed a main effect of subtype (*F*(1,43)=7.1, *p*=0.01) but no main effect of group (*F*(1,43)=0.001, *p*=0.97) and no subtype by group interaction (*F*(1,43)=0.33, *p*=0.57). Subsequent post-hoc pairwise comparisons revealed lower subgenual cingulate-amygdala connectivity in the melancholic rMDD group (M=0.12, SD=0.17) compared to the non-melancholic rMDD group (M=0.25, SD=0.11, *p*=0.01, mean difference=-0.12, 95% CI [-0.21,-0.03]). These results suggest the network of lower functional connectivity we previously reported in the melancholic rMDD patients is independent of vulnerability or resilience to recurring MDEs, which is in keeping with our original interpretation of subgenual cingulate-amygdala functional disconnection as a primary vulnerability factor for melancholia.

***Supplementary Table S1.*** *Inter-rater reliability for the SCID-I, MADRS, and PSR scales*

|  | **SCID-I subtype** | **Current MADRS** | **MADRS**  **previous MDE** | **Current PSR** | **Highest PSR during follow-up** |
| --- | --- | --- | --- | --- | --- |
| Raters | Kappa Value | ICC Value | ICC Value | ICC Value | ICC Value |
| RZ & KL | 0.60 | 0.63 | 0.45 | 0.96 | 0.98 |
| RZ & JG | – | 0.91 | 0.80 | – | – |
| KL & JG | 1.00 | 0.86 | 0.80 | – | – |
| KL & CW | – | – | – | 0.96 | 0.99 |
| **Mean** | 0.80 | 0.80 | 0.68 | 0.96 | 0.98 |

Reliability for the SCID-I mood disorders module subtype diagnosis is given as a kappa value. Reliability for the MADRS and PSR are given as intra-class correlation (ICC) values (two-way mixed with absolute agreement). RZ, KL, and JG completed the recommended training for the SCID-I for DSM-IV-TR, and RZ, KL, and CW completed the recommended training for the PSR. The SCID-I was modified to allow lifetime diagnoses of MDD subtypes, including melancholic and atypical specifiers. The MADRS was used to assess depression severity at the time of the clinical interview, and was modified to allow for retrospective assessment of the last and most severe MDE. The PSR was used to assess the severity of and impairment caused by depressive symptoms present at each follow up interview and retrospectively throughout the follow up period. The Kappa values for the SCID-I subtype diagnoses reflect moderate to perfect agreement , and ICC values for the MADRS (both current and previous MDE) and PSR reflect moderate to excellent agreement . ICC, intra-class correlation; MADRS, Montgomery-Åsberg Depression Rating Scale; MDD, major depressive disorder; MDE, major depressive episode; PSR, Psychiatric Status Rating; SCID-I, Structured Clinical Interview-I.

***Supplementary Table S2.*** Reasons for exclusion of volunteers from the current study

| **Reasons for Exclusion** | **N** |
| --- | --- |
| *Telephone Screening* | |
| MRI contraindications | 77 |
| Psychiatric disorders other than MDD | 54 |
| Current antidepressants or other centrally active medications | 52 |
| Withdrawal after telephone screening | 33 |
| Not meeting full screening criteria for MDD | 30 |
| Family history of MDD/bipolar/schizophrenia (HC group) | 26 |
| Substance or alcohol abuse | 23 |
| Current antihypertensive or statin medications | 20 |
| Left-handed | 20 |
| Non-native English speaker | 19 |
| Thyroid function problems | 19 |
| Fulfilling criteria for current MDD | 13 |
| History of cancer | 7 |
| Not remitted for long enough (>6 months) | 7 |
| Epilepsy | 5 |
| No reason recorded | 5 |
| Other general medical conditions | 5 |
| Diabetes | 4 |
| Out of age range (18 – 65 years) | 4 |
| Excluded because of age-matching (HC group) | 3 |
| Multiple sclerosis | 3 |
| History of stroke | 1 |
| Vitamin D deficiency | 1 |
| Total excluded after the telephone screening | 431 / 707 |
| *Clinical Interview (remitted MDD patients)* | |
| Unable to schedule for additional visits | 10 |
| Fulfilling criteria for a bipolar disorder | 6 |
| Fulfilling criteria for current social anxiety disorder | 6 |
| Not meeting full criteria for MDD | 5 |
| Fulfilling criteria for past substance abuse | 4 |
| Not remitted for long enough (>6 months) | 3 |
| Residual symptoms of post-traumatic stress disorder | 3 |
| Probable personality disorders | 2 |
| Fulfilling criteria for current generalized anxiety disorder | 1 |
| MRI contraindications | 1 |
| Withdrawal after the clinical interview | 1 |
| Total number of remitted MDD patients excluded after the clinical interview | 42 / 138 |
| *Clinical Interview (HC group)* | |
| Unable to schedule for additional visits | 6 |
| Probable or definite positive first degree family history of MDD | 4 |
| Fulfilling criteria for a past MDE lasting less than two months | 1 |
| Fulfilling criteria for current adjustment disorder | 1 |
| Fulfilling criteria for current MDD | 1 |
| Fulfilling criteria for current social anxiety disorder | 1 |
| Non-native English speaker | 1 |
| Past depressive episode not fulfilling criteria for a past MDE | 1 |
| Total number of HC participants excluded after the clinical interview | 16 / 64 |

Of the 707 volunteers who completed the telephone screening, 276 were eligible (184 remitted MDD patients, 92 HC participants). Of these, 202 participants agreed to complete the clinical interview after having reviewed the study’s participant information sheet (138 remitted MDD patients, 64 HC participants). Following the clinical interview, 144 participants were eligible to complete the remaining study visits (96 remitted MDD patients, 48 HC participants). Of these, 102 participants underwent resting-state fMRI scanning (63 remitted MDD patients, 39 HC participants). fMRI, functional magnetic resonance imaging; HC, healthy control; MDD, major depressive disorder; MDE, major depressive episode.

**REFERENCES**

**Fleiss, JL** (1986). *The Design and Analysis of Clinical Experiments*. Wiley: New York.

**Landis, JR & Koch, GG** (1977). The measurement of observer agreement for categorical data. *Biometrics* **33**, 159–174.

**Workman, CI, Lythe, KE, McKie, S, Moll, J, Gethin, JA, Deakin, JFW, Elliott, R & Zahn, R** (2016). Subgenual cingulate-amygdala functional disconnection and vulnerability to melancholic depression. *Neuropsychopharmacology* **41**, 2082–2090.
